# Chemistry as Exemplifying the Wisdom and Beneficence of God

**Published:** 1844-10-01

**Authors:** 


### Chemistry, as exemplifying the Wisdom and Beneficence of God. By *G. Fownes*, Ph. D., &c. London, 1844.

We are informed in the preface to this work, that in the year 1838 Mrs. Hannah Acton, widow of the late Samuel Acton, Esq., from motives of respect for the memory of her late husband, and in order to carry into effect his desire and intention, caused an investment to be made of the sum of one thousand pounds in the three per cent. Consol. Bank Annuities, in the names of the Trustees of the Royal Institution of Great Britain, the intent of which was to be devoted to the formation of a fund, out of which the sum of one hundred guineas was to be paid septennially as a prize to the person who should have been the author of the best essay illustrative of the wisdom and beneficence of the Almighty in such department of science as the committee of managers should have selected. The subject chosen for the prize of the first period of seven years was "Chemistry, as exemplifying the Wisdom and Beneficence of God." The author of the present work obtained this prize, and has now published the Essay in its present form. The object of the Essay is analogous to that of the Bridgewater Treatises, *scil.* to investigate the relations which connect man with his Creator in the noble exercise of human reason. The Being who bestowed on man this faculty, must have intended that he should acquire, through its means, some insight into the order and arrangement of creation, and some knowledge of the divine attributes. Nothing can contribute to impart this knowledge more effectually than investigating the studied arrangements, the preconcerted adaptations, the multiplied evidences of intention, the signal proofs of beneficent design which pervade the entire series of terrestrial beings.

In discussing his subject, the author has adopted the following order :—

*The Chemical History of the Earth, and the Atmosphere.*
*The Peculiarities which characterize Organic Substances generally.*
*The Composition and Sustenance of Plants.*
*The Relations existing between Plants and Animals.*

In the division of the undecompounded substances, otherwise called "simple" or "elementary substances," being the fifty-five distinct forms or modifications of matter brought to light by the researches of modern chemistry, the author adopts the arrangement into metallic, and non-metallic substances. At the head of the latter class stand oxygen, chlorine, iodine, sulphur, carbon, hydrogen, &c.—substances of the very highest importance in the great scheme of Nature, from the universality of their occurrence, and their intimate connexion with all the phenomena of organic life. This division, however, he acknowledges to be more convenient in practice than correct in principle, as a regular gradation from the one class to the other can be easily traced. Of these fifty-five elements, some eight or ten will form the whole list of substances concerned in the formation of the great bulk and mass of all those objects we see around us. The atmosphere is made up of two—viz. oxygen and nitrogen, with mere traces of two others, carbon and hydrogen. Two of these again, oxygen and hydrogen, by their chemical union, give rise to water, occupying in the shape of ocean of unknown depth, lakes and rivers, nearly three-fourths of the surface of our planet; the solid earth is chiefly made up of the oxides of one non-metallic body, silicon, and two metals, aluminium and calcium. To these may be added potassium, whose oxide is a constituent of felspar, the characteristic ingredient of granite; sodium, of which the water of the ocean is the great depository; and iron, whose oxide is to be found everywhere to a greater or less extent. These substances really include all the known components of the earth. Other substances, such as magnesium in a state of oxide; sulphur in a state of gypsum, and in union with certain metals, are found here and there, in local deposits of considerable extent, while the remainder are still more limited in their distribution. In the organic world the same, or still greater simplicity is observed; carbon, hydrogen, oxygen, and nitrogen, a little phosphorus and sulphur, and sometimes a small proportion of two or three alkaline and earthy salts, are the sole materials which have been employed in the construction of those countless multitudes of *orders* of living objects which people the earth, and clothe it with beauty. This apparent paucity of original materials is, however, more than compensated by the very peculiar properties of some of those selected. Carbon, hydrogen, oxygen, and nitrogen, are distinguished above all other bodies by the innumerable compounds they are capable of forming, by union among themselves. Modern organic chemistry, vast as it already is, consists of little more than the study of these four elements and their combinations. Geology is happily brought into requisition on the occasion, in pointing out the advantages which mankind derive from the particular mode of structure, or arrangement, of the beds and masses of rock which form the solid earth. The evident design and contrivance exhibited in the disposition of our mineral treasures must convince the mind of forethought and provision for man's necessities and comfort. We see in the English coal-field excellent iron-ore lying interstratified with the fuel necessary to reduce it; we observe the lime-stone, used as a flux, and even the very grit and fire-clay to build the furnace, are all to be found in one and the same series. This curious relation of the ore and the fuel enables us to obtain at a cheap rate this noblest of all the metals, which is applied to innumerable purposes of daily life and convenience. Again, the author points to the mountains of Sweden and Norway, which contain inexhaustible beds of magnetic iron-ore, which, when reduced by charcoal, yields the finest and purest bar-iron for making steel. Now wood-charcoal being the only fuel at all fit for employment on this occasion, forests of pines have been accordingly planted by the provident hand of Nature in these desolate regions, by means of which the iron-maker is enabled to pursue his labours. As another instance of prospective contrivance, the author points to the material constituting the great mountain chains which ridge and furrow the earth's surface, and upon whose flanks and lower slopes rest the different stratified aqueous deposits of conglomerate, and slate, and sand-stone, and calcareous matter, great part of which have been directly derived from the mechanical degradation of the central mountain nucleus itself—this material is granite, which has been, as it were, deliberately chosen from a previous knowledge of its peculiar chemical fitness for the office in question. It is obvious that to animal existence a supply of food is indispensable, which must be derived either directly or indirectly from the vegetable kingdom; animal life therefore presupposes vegetation. Now besides the organic constituents, as carbon, hydrogen, oxygen, and nitrogen, which plants derive from the atmosphere, they are also known to contain, in every case and without a single exception, other substances equally indispensable to vegetable life; these substances are the alkalis, potash and soda, phosphoric and sulphuric acids, lime and magnesia, silica, &c.; these form the contingent of the food really furnished by the soil in which the plant grows, and are just as necessary to its well-being as the carbonic acid, or the water it gets from the air. The most indispensable of these principles to land-vegetation is potash; another, perhaps equally important substance, is phosphoric acid, a body as universally diffused in the vegetable kingdom as potash itself. The reason of this provision will at once appear, if we recollect the important function fulfilled by one of the earthy phosphates in the animal economy, viz. the phosphate of lime, which gives strength to the bony frame-work of the animal body. Now the source of this phosphorus, there is every reason to think, is to be found in the antient massive granite already spoken of as forming the basis of our present system of continents, and there cannot be a doubt that the origin of potash is from the same source; so that it cannot be said, that it is by blind chance that granite occupies so important a place in the frame-work of the earth.

The author next directs attention to the chemical study of the atmosphere. This is known to consist of four distinct elementary substances, oxygen, hydrogen, nitrogen, and carbon, *mixed* in certain proportions, which have been found to be invariable for air obtained from every region of the earth. Now to what is this extraordinary uniformity of mixture throughout the whole extent of the atmosphere, its constituents being merely mixed, to be attributed? The several constituents differ in specific gravity; and hence one would suppose that they would arrange themselves in the order of their densities. The answer involves the principle of gaseous diffusion. The advantages resulting from this principle are incalculable. There are processes constantly going on around us in which gaseous matter and vapours, prejudicial in the highest degree to animal life, are unceasingly evolved; the function of respiration, the burning of wood and coal for fuel, are attended with the conversion of the free oxygen of the air into carbonic acid. By the putrefactive decomposition of animal and vegetable substances, poisonous principles, far worse than carbonic acid, are given off into the air. If the heavy carbonic acid, so copiously generated from the crowding together of multitudes of men into towns and cities, were simply to obey the natural law of gravitation, and spread itself out upon the surface in such localities, a state of things would arise absolutely unendurable: instantaneous death would be the inevitable result to the unfortunate beings who should be placed beneath such a stratum of carbonic acid. Such a state of things, however, it may be said, and truly said, is impossible. Carbonic acid is freely soluble in water—the water of the sea would dissolve this gaseous stratum and so effect its total disappearance, so that man would have no reason to dread asphyxia by carbonic acid. Still, such a mode of removing the carbonic acid from the atmosphere would be fatal to vegetable life, as it is well known that plants depend, in a very great measure, upon the carbonic acid of the atmosphere for their sustenance, which acid they decompose in order to obtain carbon.

The advantages resulting from this law of gaseous diffusion is equally obvious with respect to oxygen and nitrogen. If instead of being uniformly mixed, as now, whereby the chemical energies of the one become modified and, as it were, diluted by the other, these two gases were to form two immense layers of unequal thickness arranged in the order of their densities, animal existence would be entirely out of the question, an atmosphere of pure oxygen being just as fatal to life as one destitute of that element. The equable diffusion of the aqueous vapour contained in the atmosphere is no less important. In many warm countries, during a great part of the year, rain seldom or never falls, and it is only from the copious dews deposited in the night that vegetables derive the supply of moisture required for their growth, and to sustain them, by the cooling effects of evaporation, from the scorching rays of the noon-day sun.

Our author next comes to the *Chemistry of Organization,* and endeavours to establish the proposition that "the selection out of the whole number of elementary substances of the four organic elements—carbon, hydrogen, oxygen, and nitrogen—for the purposes of organization, was expressly made from a full previous knowledge of the very extraordinary chemical properties possessed by these substances by which they alone are fitted for the objects intended." Design, therefore, is proved. There is, we must observe, in this part of our author's essay, a something wanted to show *why* these four elementary substances are the fittest materials for the construction of organized beings. Does he mean that they are so by reason of the instability and want of permanency of the combinations into which they enter in the formation of organised beings, an instability necessary for the purpose of allowing the various changes which take place in such beings?

Our author next considers the subject of *Vegetable Chemistry.* He divides the proximate vegetable principles into two classes—viz. those containing nitrogen, and those destitute of that element. The principal groups of substances contained in the latter are the following;—1. Saccharine and amylaceous bodies. 2. Vegetable acids. 3. Fatty and resinous principles. The sweet principles of plants are somewhat numerous. The chief modifications of them are the sugar of the cane, and the sugar of grapes—the sugar of the cane is prepared from the juice of the sugar cane in most of the warmer regions of the earth. Its most refined form is loaf-sugar or white candy—the most obvious properties of this substance are its free solubility in water, pure sweet taste. Grape sugar, the ordinary sweet principle of ripe fruits, is readily distinguished from the other kinds; it crystallizes from its watery solution slowly and with difficulty—is much less soluble than cane sugar, and very inferior in sweetness—is a much more stable and permanent substance than the cane-sugar, and probably has a simpler chemical constitution.

*Starch or Fecula* is a body of great interest, from its universal occurrence in the vegetable kingdom, and the important objects it there fulfills. When starch is put into cold water and gently heated, its granules break, and the whole becomes a gelatinous mass, freely miscible with water. This change is accounted for thus: the grains of the starch consist of a soluble matter enveloped in a fine membrane, which is rent at a high temperature, and the internal substance attacked ; the internal substance thus dissolved is called " amidine." When thick gelatinous starch is boiled for a few minutes with any dilute acid it changes to a fluid limpid as water, and, by removing the acid and evaporating to dryness, a gummy substance is obtained to which the name of " dextrine" has been given. By continuing the boiling for several hours, and then separating the acid and boiling down the clear solution to a small bulk, we get a syrupy liquid, very sweet to the taste, which, on standing for a few days, entirely solidifies into a mass of grape sugar, exceeding in weight the starch from which it was produced. In these transformations the acid is not acted on—the whole affair lies between the amidine and the elements of water, grape-sugar containing more oxygen and hydrogen, compared with the quantity of carbon, than starch. The same effect will be produced by using a solution of a little common malt instead of the dilute acid. The conversion of the starch into sugar is supposed in this case to be due to the presence of a substance in the malt, called " diastase," imagined to exist in all seeds under similar circumstances—it seems to belong to the class of vegetable albuminous principles. What has been now stated will suffice to render intelligible the beautiful order of arrangements by which plants are furnished with a sudden and copious supply of already assimilated food at certain particular periods of their life, when the drain upon the natural powers is excessive, or when the plant, from its feeble and undeveloped state, is unable to obtain and digest food from without. It is well known that a very large proportion of vegetables, in the temperate zones, at the close of every Summer, either perish completely, or die down to the roots, which remain in the ground till the following Spring. This is sometimes attributed to the influence of cold. A more efficient cause is, however, to be found in the plant itself, whose death is the result of the exhausting nature of the process by which provision is made for another generation of similar beings. The period of inflorescence and seed-bearing are attended with a large expenditure of costly material. In the formation of the fruit and seeds soluble gummy and saccharine matters are conveyed to those points and there fixed, to the manifest detriment of the whole system, which suffers by the withdrawal of these substances. Now, in order to guard against the evil consequences of a necessary function, Nature usually employs the time previous to flowering, when the vegetative power is most active, in storing up, in different parts of the plant, a quantity of starch, ready for use, when the pressing occasion arrives, at which time it is re-dissolved, by the aid of diastase perhaps, and once more added to the general stock of nutriment to promote the maturation of the seeds. These various curious processes cannot fail to exhibit to the attentive observer glimpses of a general design of the most surpassing beauty of contrivance. These, with lignin and gum, are the principal members of the very important class of neutral, non-azotized, vegetable principles. With respect to their chemical nature, they are ternary compounds of carbon, hydrogen and oxygen, the two latter elements almost invariably existing in the proportions to form water. This is one character of the group—another is, their disinclination to form chemical compounds with other substances—they do, however, occasionally so combine, but with very feeble energy. The vegetable acids occur, often in large quantities, throughout the whole vegetable kingdom—they seldom occur in a free state—their most usual condition is in that of a salt of an alkali, or earth, or sometimes an organic alkaline principle; a salt of potash, with excess of acid, is very common. With respect to chemical composition, the vegetable acids usually exhibit a larger comparative quantity of oxygen than the other proximate principles. They are destitute of azote. Oils and fats form an important series of bodies characterized, in a chemical point of view, by a deficiency or total absence of oxygen, with which circumstance their combustible character is connected. Seeds are very often loaded with oil or soft fat, which may be extracted by simple pressure, or by heating with water. In close connexion with the (volatile) oils are the resins, which are looked on as oxidized volatile oils. The vegetable principles containing nitrogen have been subdivided into VEGETO-ALKALIS, and the ALBUMINOUS PRINCIPLES. The latter being the more important, the author considers at some length. The change by which sugar becomes converted into spirit is brought about by the agency of some albuminous principle, the presence of azotized organic matter of this peculiar kind being absolutely a necessary condition for the occurrence of vinous fermentation. To this change all saccharine liquids are subject, provided they contain in solution azotized matter capable of putrefying, and are placed in circumstances favourable to that event. The azotized principle runs into spontaneous decay; it is slowly resolved into carbonic acid, ammonia and other products, and the molecular disturbance then produced in the decomposing body or "ferment," is propagated to the sugar, the equilibrium of opposing attractions among the then elements of that substance is overthrown, while new and more stable compounds arise in place of that destroyed. Conversion into alcohol is not the only change which sugar is susceptible of undergoing under the influence of a ferment. It may also pass into an acid state, producing lactic acid; or it may be converted into the sweet principle of manna. The kind of change seems to depend on the particular stage of decomposition of the ferment itself. If the mysteries of vital chemistry are fated ever to be unravelled, it will be by the careful investigation of re-actions such as these. The decay of organic tissues in the bodies of plants and animals, is a process which has for its object not only the removal of useless matter, but its conversion into a form once more capable of supporting life. It may be observed that reproduction and decay follow each other's footsteps throughout the whole system of Nature, and that the conditions of life and death are mutually involved in, and dependent upon each other. The great agent in all these matters is the oxygen of the air, and the ultimate result is the transformation of the organic substance into such bodies as carbonic acid, water, carburetted hydrogen, ammonia, or nitric acid. The intermediate states, passed through, which are very numerous, probably resemble those through which sugar passes. The never-ceasing activity of free oxygen is thus not only the main spring of life, but it is the cleanser, the purifier of earth and air and sea, from the defilements constantly poured upon, and into these latter, from the countless sources of poisonous contamination around us. Vapours of volatile substances, rich in hydrogen, the putrid effluvia of pestilence, become gradually destroyed by a real process of burning, more slowly, but not less completely, than in the flame of a furnace. The water of lakes and rivers, and of the great ocean itself, owes its sweetness to the same cause. In the remaining part of this chapter the author enters somewhat in detail into the establishment of the proposition that adult plants live upon the air; that they get their carbon from its carbonic acid, their hydrogen from its moisture, and their nitrogen from the little ammoniacal vapour which there exists. Thus plants feed upon inorganic substances. The various processes of the nutrition of plants give incontestible proofs of design and adaptation of the most general and comprehensive character.

He next takes up the subject of Animal Chemistry, as affording further proofs of the same *Design*. But as we find our author gives here little else than Liebig's theories, and as a complete analysis of these has already appeared in the pages of this Journal, and a somewhat summary refutation of some of them may be found in the last number of our Journal, more especially of the combustion-theory, we shall close our analysis of this truly interesting little work, from the perusal of which we have derived both pleasure and instruction.

